# Molecular etiology and genotype-phenotype correlation of Chinese Han deaf patients with type I and type II Waardenburg Syndrome

**DOI:** 10.1038/srep35498

**Published:** 2016-10-19

**Authors:** Lianhua Sun, Xiaohua Li, Jun Shi, Xiuhong Pang, Yechen Hu, Xiaowen Wang, Hao Wu, Tao Yang

**Affiliations:** 1 Department of Otolaryngology–Head and Neck Surgery, Shanghai Ninth People’s Hospital, Shanghai Jiaotong University School of Medicine, Shanghai, China; 2 Ear Institute, Shanghai Jiaotong University School of Medicine, Shanghai, China; 3 Shanghai Key Laboratory of Translational Medicine on Ear and Nose Diseases, Shanghai, China; 4 Department of Otorhinolaryngology-Head and Neck Surgery, Xinhua Hospital, Shanghai Jiaotong University School of Medicine, Shanghai, China; 5 Department of Otology, the First Affiliated Hospital of Zhengzhou University, Zhengzhou, Henan, China; 6 Department of Otorhinolaryngology-Head and Neck Surgery, Taizhou People’s Hospital, Jiangsu Province, China; 7 High School Affiliated to Fudan University, Shanghai, China

## Abstract

Waardenburg syndrome (WS) characterized by sensorineural hearing loss and pigmentary abnormalities is genetically heterogeneous and phenotypically variable. This study investigated the molecular etiology and genotype-phenotype correlation of WS in 36 Chinese Han deaf probands and 16 additional family members that were clinically diagnosed with WS type I (WS1, n = 8) and type II (WS2, n = 42). Mutation screening of six WS-associated genes detected *PAX3* mutations in 6 (86%) of the 7 WS1 probands. Among the 29 WS2 probands, 13 (45%) and 10 (34%) were identified with *SOX10* and *MITF* mutations, respectively. Nineteen of the 26 detected mutations were novel. In WS2 probands whose parental DNA samples were available, *de novo* mutations were frequently seen for *SOX10* mutations (7/8) but not for *MITF* mutations (0/5, *P* = 0.005). Excessive freckle, a common feature of WS2 in Chinese Hans, was frequent in WS2 probands with *MITF* mutations (7/10) but not in those with *SOX10* mutations (0/13, *P* = 4.9 × 10^−4^). Our results showed that mutations in *SOX10* and *MITF* are two major causes for deafness associated with WS2. These two subtypes of WS2 can be distinguished by the high *de novo* rate of the *SOX10* mutations and the excessive freckle phenotype exclusively associated with the *MITF* mutations.

Waardenburg Syndrome (WS) is relatively common among syndromic deafness, with an estimated prevalence of 1 in 42000 in the general population and 1–3% among the congenitally deaf^1^. It is mainly characterized by sensorineural deafness and various types of pigmentary abnormalities including heterochromic iridis, patchy de-pigmentation of the skin and premature graying of the hair. Based on the additional symptoms, WS can be further categorized into WS type I with dystrophia canthorum (WS1, OMIM193500), WS type II without additional symptoms (WS2, OMIM 193510, 600193, 606662, 608890 and 611584), WS type III with dystrophia canthorum and upper limb anomalies (WS3, also called Klein-Waardenburg syndrome, OMIM148820) and WS type IV with aganglionic megacolon (WS4, also called Shah-Waardenburg Syndrome or Waardenburg-Hirschsprung Disease, OMIM 277580, 613265 and 613266). In many cases, the clinical phenotypes of WS are incompletely penetrant. Hearing loss, for example, was estimated to occur in 60% for WS1, 90% for WS2, but only 5% for WS4^2^,^3^. Variable phenotypic expression can be observed both interfamilially and intrafamilially, suggesting of interplay between genetic modifiers and environmental factors.

To date, six causative genes have been identified for WS, including *PAX3* encoding the paired box 3 transcription factor, *MITF* encoding the microphthalmia-associated transcription factor, *SOX10* encoding the SRY (sex determining region Y) box 10 transcription factor, *SNAI2* encoding the snail homolog 2 transcription factor, *EDN3* encoding the endothelin-3 and *EDNRB* encoding the endothelin receptor type B. Most cases of WS1 and some moderate cases of WS3 are caused by heterozygous mutations in *PAX3*, while homozygous or compound heterozygous mutations of *PAX3* have been identified in some severe cases of WS3^4^. In WS2, heterozygous mutations in *MITF* and *SOX10* are estimated to account for 15% of cases each, while heterozygous mutations in *EDNRB* and homozygous mutations in *SNAI2* haven been identified in less than 5%^5^. Two additional WS2 loci, WS2B (OMIM 600193) and WS2C (OMIM 606662), have been reported without pathogenic genes identified^6^. In WS4, Approximately 50% of cases are due to heterozygous mutations in *SOX10* and 20–30% to homozygous or heterozygous mutations in *EDNRB* and *EDN3*.

WS1 and WS2 were two major subtypes of WS associated with hearing loss. Unlike the former, WS2 is genetically heterogeneous and so far the genotype-phenotype correlation of the WS2 genes remains unclear. In this study, 50 Chinese Han deaf patients with WS type I and II were clinically characterized and genetically screened for mutations in *PAX3*, *MITF*, *SOX10*, *SNAI*, *EDN3* and *EDNRB*. Our results showed that heterozygous mutations in *MITF* and *SOX10*, two major causes of WS2, have distinguishable *de novo* rates and clinical features.

## Results

### Clinical characteristics of the WS patients

The clinical features of the WS probands were summarized in Table 1. All 36 probands have sensorineural, severe-to-profound hearing loss and at least one type of pigmentary abnormalities including heterochromic iridis (n = 34), excessive freckle (n = 7), patchy de-pigmentation of the skin (n = 6) and premature graying of the hair (n = 6). No musculoskeletal anomaly or intestinal aganglionosis was observed in any of the probands. Based on the W-indexes, 7 probands were diagnosed with WS1 (W-indexes > 2.10) and 29 with WS2 (W-indexes <1.90).

**Figure 1:**
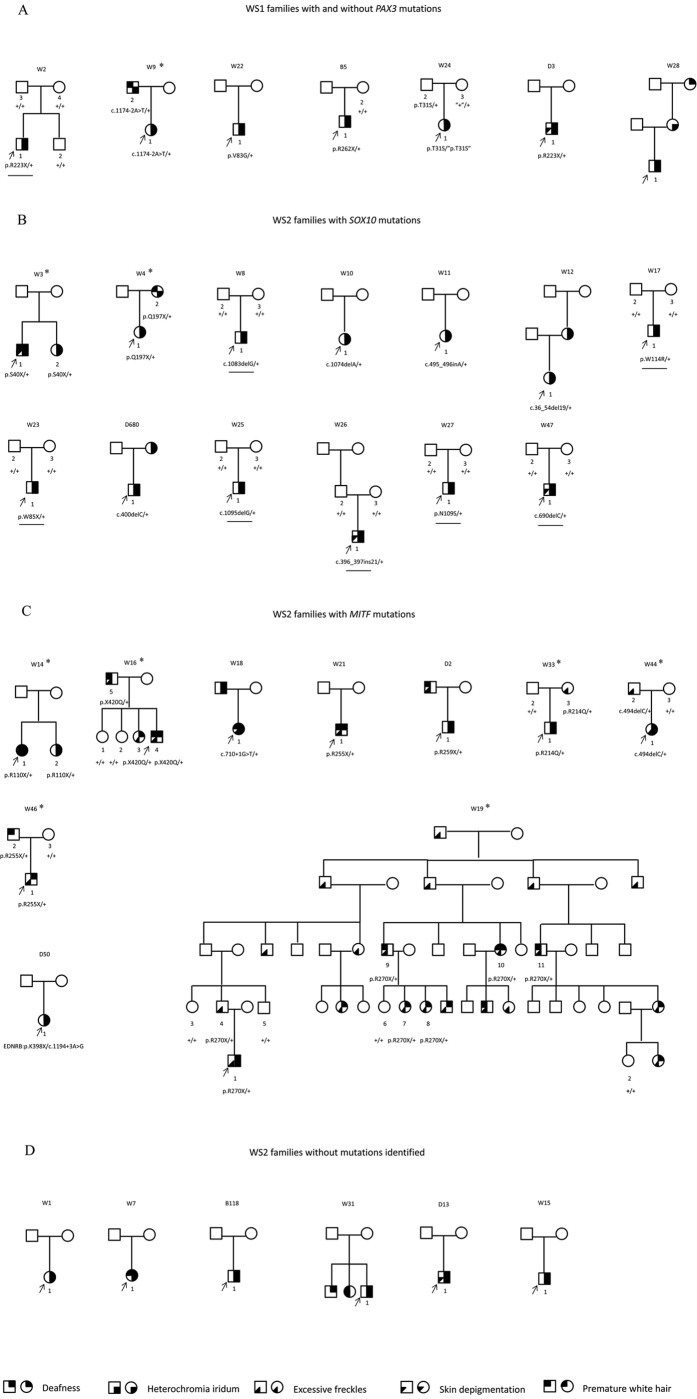
Pedigrees of the WS patients showing their phenotypes and genotypes. Individuals with a number assigned participated in the current study. Phenotypes of the rest of the family members were based on relative’s description. The probands were pointed by arrows. Families with additional affected members who participated in the current study were marked by asterisks. (**A**) WS1 families with and without *PAX3* mutations. Note in family W24, the apparent non-segregation of the p.T31S variant (marked by quotation marks) indicated a likely gross deletion in *PAX3* in individual W24-3 and W24-1. (**B**) WS2 families with *SOX10* mutations. The *de novo* mutations were underlined. (**C**) WS2 families with MITF mutations. (**D**) WS2 families without mutation identified.

Intrafamilial varieties of the WS-associated phenotypes can be observed in all of the nine families with additional affected members (Fig. 1, marked by asterisks). Among them, heterochromic iridis is the most consistent intrafamilial phenotype (4/7), followed by excessive freckle (3/5), hearing loss (2/9), patchy de-pigmentation of the skin (0/2) and premature graying of the hair (0/6).

### Mutations identified in the WS patients

The mutations identified in the WS patients were summarized in Table 2 and labeled in Fig. 1. In 7 WS1 families, heterozygous mutations in *PAX3* were identified in 5 of them. In addition, apparent haplotype non-segregation of a *PAX3* p.T31S variant in Family W24 suggested the presence of a gross deletion in *PAX3* in W24-1 and W24-3 (Fig. 1A). In 29 WS2 families, heterozygous mutations in *SOX10* and *MITF* were identified in 13 and 10 of them, respectively (Fig. 1B,C). Seven of the 26 different mutations reported in this study were previously reported and 19 were novel (not seen in ExAC and 1000Genomes database). The majority (20/27) of the mutations were truncating or null mutations including nonsense mutations (n = 9), frameshifting indels (n = 8) and splicing site mutations (n = 2) and gross deletion (n = 1). They were predicted to lead to null alleles, prematurely stopped protein products or nonsense-mediate decay of the mRNA. The 4 missense mutations identified in this study all changed an evolutionarily conserved amino acid (Phylop scores >4.6) and were predicted to be disease-causing by all six computational tools Mutation Taster, Polyphen-2, MetaSVM, PROVEAN, SIFT and CADD (Table 3). The rest of the two mutations were a non-frameshifting insertion resulting in extra 7 amino acids in SOX10 and a non-stop mutation resulting in extra 51 amino acids in the C-terminus of MITF. None of the 26 mutations were seen in 300 ethnically matched normal hearing controls.

### *De novo* rates of the *SOX10* and *MITF* mutations in WS2 probands

Parental blood DNA samples were available for 13 WS2 probands with *SOX10* (n = 8) and *MITF* (n = 5) mutations. For 7 of the 8 WS2 probands with *SOX10* mutations, the corresponding mutations were not detected in either of the parents, suggesting that the mutations occurred *de novo* (Fig. 1B, *de novo* mutations were underlined). In contrast, for all 5 WS2 probands with *MITF* mutations, the corresponding mutations can be detected in one of the parents (Fig. 1C). The difference of *de novo* rates between *SOX10* and *MITF* mutations was statistically significant (*P* = 0.005).

### Genotype-phenotype correlation of the *SOX10* and *MITF* mutations in WS2

The pigmentary phenotypes of the WS2 probands with *SOX10* (n = 13) and *MITF* (n = 10) mutations were compared in Table 4. While the percentages of WS2 probands with heterochromic iridis, premature graying of the hair or patchy de-pigmentation of the skin were not significantly different between those with *SOX10* and *MITF* mutations (*P *> 0.05), excessive freckle (as shown in previous reports^7^,^8^), a common WS2 phenotype in Chinese Han patients, was frequent in WS2 probands with *MITF* mutations (7/10) but absent in those with *SOX10* mutations (0/13, *P* = 4.9 × 10^−4^). Logistic regression analysis showed that this excessive freckle phenotype was not affected by other confounding factors such as gender or age of the patients (*P* = 0.8129 and 0.1559, respectively, Supplementary Table S1).

## Discussion

In this study, we aimed to explore the molecular etiology and genotype-phenotype correlation in deaf patients associated with WS. Among 36 probands, 7 and 29 can be classified as WS1 and WS2, respectively, based on their clinical phenotype (Table 1) and molecular diagnosis (Table 2), showing that WS1 and WS2 were two major WS subtypes associated with hearing loss. While mutations in *PAX3* were the major cause for WS1 (6/7), the molecular etiology of WS2 was heterogeneous and attributable to two major causative genes *SOX10* (13/29) and *MITF* (10/29).

By parental genotyping, we revealed an interesting inheritance pattern in WS2, as *de novo* mutations were frequently found in WS2 probands with *SOX10* mutations (7/8) but not in those with *MITF* mutations (0/5, *P* = 0.005). Notably, no evidence suggested that the seven *de novo* mutations in *SOX10* were from mutation hot-spots, as they occurred at different nucleotide positions and six of them were not previously reported (Table 2). The high *de novo* rates of *SOX10* mutations in WS2 may need special attention during the course of genetic diagnosis and counseling, as it can be initially mistaken as recessive inheritance prior to testing or be interpreted with over-estimated recurrent risk without further parental testing.

Consistent with its pathogenic role in WS2, *MITF* encodes a basic helix-loop-helix, leucine zipper transcription factor that plays a critical function in survival and differentiation of melanocytes that produce melanin pigments^9^. SOX10, the SRY-related transcription factor, binds to *MITF* promoter and directly activates MITF’s expression^10^. Mutations in those two genes, therefore, are likely involved in the pathogenesis of WS2 through the same pathway and produce similar clinical phenotypes. Based on previous reports, the clinical features of WS2 were indeed indistinguishable between that resulted from *SOX10* and *MITF* mutations^11^. In this study, we compared the pigmentary abnormalities between WS2 probands with *SOX10* and *MITF* mutations (Table 4). Both previous reports and the present study showed that excessive freckle was frequently observed in Chinese Han WS patients^7^. Interestingly, this special subtype of cutaneous pigmentary disturbance appeared to be unique for WS2 probands with *MITF* mutations (7/10) but was absent in those with *SOX10* mutations (0/13, *P* = 4.9 × 10^−4^). To our knowledge, this is the first report showing the clinical differences between WS2 patients with *SOX10* and *MITF* mutations.

In conclusion, our study revealed that WS2 due to *SOX10* and *MITF* mutations have discrepant *de novo* mutation rates and distinguishable clinical feature in excessive freckle. These new findings may facilitate precise diagnosis and genetic counseling of the heterogeneous WS2.

## Methods and Materials

### Patients

Thirty-six Chinese Han deaf probands clinically diagnosed with WS were recruited through their visit to the Department of Otolaryngology-Head and Neck Surgery, Xinhua Hospital, Shanghai, China. Fourteen of them reported family history of WS features including hearing impairment, heterochromia iridis, premature graying of the hair, excessive freckle and patchy de-pigmentation of the skin. A total of 16 additional affected members from 9 families (marked by asterisks in Fig. 1) were subsequently recruited into this study. All patients or guardians gave written, informed consent to participate in this study. This study was approved by the Ethics Committee of Xinhua Hospital, Shanghai Jiaotong University School of Medicine and was in compliance with the Declaration of Helsinki.

### Clinical evaluations

Comprehensive auditory, ophthalmologic, dermatologic and neurological examinations were performed on all subjects. Auditory evaluations included otoscope examination, tympanometry, pure-tone audiometry (PTA) and/or auditory brainstem response (ABR, used for subjects with very young age). Degree of hearing impairment was calculated as the average of the hearing levels at 0.5, 1.0, 2.0 and 4.0 KHz for the better ear. The severity of hearing impairment was defined as mild (20–40 dB), moderate (41–70 dB), severe (71–95 dB) and profound (>95 dB). Special attention was given to the pigmentary abnormalities of iris, skin and hair as well as the developmental defects including dystopia canthorum, limb abnormalities and intestinal aganglionosis. W-index, the biometric index of dystopia canthorum was measured as previously described^1^. The patients were categorized into subtypes of WS according to the criteria proposed by the WS consortium^1^.

### Mutation analysis

Genomic DNA was extracted from peripheral blood samples using the Blood DNA kit (TIANGEN Biotech, Beijing, China). Mutation screening of *PAX3*, *MITF*, *SOX10*, *SNAI2*, *EDN3* and *EDNRB* was performed by polymerase chain reaction (PCR) amplification and sequencing of all exons and flanking splicing sites. Possible pathogenic effects of the missense mutations were evaluated by computational tools including Mutation Taster, Polyphen-2, MetaSVM, PROVEAN (cut-off score <−1.3), SIFT (cut-off score <0.05) and CADD (cut-off score >20).

### Statistical analysis

Fisher’s exact test was used to compare: 1) percentages of WS2 probands carrying *de novo* mutations between those with *MITF* and *SOX10* mutations; 2) percentages of WS2 probands exhibiting various pigmentary phenotypes between those with *MITF* and *SOX10* mutations (The *P*-value thresholds of significance were set as 0.05/4 = 0.0125 for Bonferroni correction of multiple testing in the latter). *P*-values were presented as the result of the two-tailed analysis. Logistic regression analysis was performed to test the potential confounding effects of gender and age on the excessive freckle phenotype.

## Additional Information

**How to cite this article**: Sun, L. *et al.* Molecular etiology and genotype-phenotype correlation of Chinese Han deaf patients with type I and type II Waardenburg Syndrome. *Sci. Rep.* **6**, 35498; doi: 10.1038/srep35498 (2016).

## Supplementary Material

Supplementary Information

## Figures and Tables

**Table 1:** Clinical features of the 36 WS probands.

Proband	Gender	Age (years)	Dystopia canthorum	Pigmentary abnormalities^*^	Hearing loss	WS subtype	Family history of WS
W2-1	M	9	Yes	E	Profound	WS1	No
W9-1	F	16	Yes	E	Profound	WS1	Yes
W22-1	M	5	Yes	E	Severe	WS1	No
B5-1	M	5	Yes	E	Profound	WS1	No
W24-1	F	4	Yes	E	Profound	WS1	Yes
D3-1	M	5	Yes	E+P	Profound	WS1	No
W28-1	M	1	Yes	E	Profound	WS1	Yes
W3-1	M	18	No	E+W+P	Profound	WS2	Yes
W4-1	F	13	No	E	Profound	WS2	Yes
W8-1	M	17	No	E	Profound	WS2	No
W10-1	F	19	No	E	Profound	WS2	No
W11-1	F	18	No	E	Profound	WS2	No
W12-1	F	2	No	E	Profound	WS2	Yes
W17-1	M	1	No	E	Profound	WS2	No
W23-1	M	5	No	E	Profound	WS2	No
D680-1	M	1	No	E	Profound	WS2	Yes
W25-1	M	9	No	E	Profound	WS2	No
W26-1	M	2	No	E+P	Profound	WS2	No
W27-1	M	4	No	E	Profound	WS2	No
W47-1	M	1	No	E+F	Profound	WS2	No
W14-1	F	15	No	E+W +F+P	Profound	WS2	Yes
W16-4	M	27	No	W+F	Profound	WS2	Yes
W18-1	F	31	No	E+W+F	Profound	WS2	Yes
W19-1	M	3	No	E+F	Profound	WS2	Yes
W21-1	M	31	No	W+F	Profound	WS2	No
D2-1	M	3	No	E	Profound	WS2	Yes
W33-1	M	3	No	E	Profound	WS2	Yes
W44-1	F	8	No	F	Profound	WS2	Yes
W46-1	M	6	No	E+P	Profound	WS2	Yes
D50-1	F	4	No	E	Profound	WS2	No
W1-1	F	7	No	E	Profound	WS2	No
W7-1	F	11	No	E+W	Profound	WS2	No
B118-1	M	3	No	E	Profound	WS2	No
W31-1	M	60	No	E	Profound	WS2	Yes
D13-1	M	3	No	E+P	Profound	WS2	No
W15-1	M	1	No	E	Profound	WS2	No

^*^E: heterochromiairidum; W: premature whitening of the hair; F: excessive freckles; P: Patchy skin depigmentation.

**Table 2:** Mutations identified in the WS probands.

Proband	Gene	Mutation type	Nucletide change^*^	Amino acid change	Allele frequencies in			Novelty	*De novo*
					ExAC	1000 Genomes	300 Chinese Han controls		
W2-1	*PAX3*	Nonsense	c.667C>T	p.R223X	0.000008241	0	0	Reported^1^	Yes
D3-1	*PAX3*	Nonsense	c.667C>T	p.R223X	0.000008241	0	0	Reported^1^	Unknown
B5-1	*PAX3*	Nonsense	c.784C>T	p.R262X	0	0	0	Reported^2^	Unknown
W24-1	*PAX3*	Deletion	Unspecified^**^	Unspecified^**^	0	0	0	Novel	No
W9-1	*PAX3*	Splice site	c.1174-2A>T	p.V392fs	0	0	0	Novel	No
W22-1	*PAX3*	Missense	c.248T>G	p.V83G	0	0	0	Novel	Unknown
W8-1	*SOX10*	Frameshift indel	c.1083delG	p.G362fs	0	0	0	Novel	Yes
W10-1	*SOX10*	Frameshift indel	c.1074delA	p.E359fs	0	0	0	Novel	Unknown
W11-1	*SOX10*	Frameshift indel	c.495-496insA	p.D167fs	0	0	0	Novel	Unknown
W12-1	*SOX10*	Frameshift indel	c.36-54del19bp	p.V15fs	0	0	0	Novel	Unknown
D680-1	*SOX10*	Frameshift indel	c.400delC	p.134Lfs	0	0	0	Novel	Unknown
W25-1	*SOX10*	Frameshift indel	c.1095delG	p.G366fs	0	0	0	Novel	Yes
W47-1	*SOX10*	Frameshift indel	c.690delC	p.H230fs	0	0	0	Novel	Yes
W3-1	*SOX10*	Nonsense	c.119C>A	p.S40X	0	0	0	novel	Unknown
W4-1	*SOX10*	Nonsense	c.589C>T	p.Q197X	0	0	0	novel	No
W23-1	*SOX10*	Nonsense	c.255G>A	p.W85X	0	0	0	Reported^3^	Yes
W26-1	*SOX10*	Non-frameshift indel	c.396-397ins21bp	p.132_133ins7aa	0	0	0	Novel	Yes
W17-1	*SOX10*	Missense	c.340T>C	p.W114R	0	0	0	Novel	Yes
W27-1	*SOX10*	Missense	c.326A>G	p.N109S	0	0	0	Novel	Yes
W44-1	*MITF*	Frameshift indel	c.494delC	p.P165fs	0	0	0	Novel	No
W14-1	*MITF*	Nonsense	c.328C>T	p.R110X	0	0	0	Reported^4^	No
W21-1	*MITF*	Nonsense	c.763C>T	p.R255X	0	0	0	Reported^6^	Unknown
W46-1	*MITF*	Nonsense	c.763C>T	p.R255X	0	0	0	Reported^6^	No
D2-1	*MITF*	Nonsense	c.775C>T	p.R259X	0	0	0	Reported^7^	Unknown
W19-1	*MITF*	Nonsense	c.808C>T	p.R270X	0	0	0	Novel	No
W18-1	*MITF*	Splice site	c.710+1G>T	p.P237fs	0	0	0	Reported^5^	Unknown
W16-4	*MITF*	No-stop	c.1258T>C	p.X420Qext51	0	0	0	Novel	No
W33-1	*MITF*	Missense	c.641G>A	p.R214Q	0	0	0	Novel	No

^*^The referenced sequences are NM_181459 for *PAX3*, NM_006941 for *SOX10* and NM_000248 for *MITF*.

^**^Suggested by haplotype non-segregation of a *PAX3* p.T31S variant in Family W24.

**Table 3:** Computational analysis of the missense mutations identified in the WS probands.

Gene	Mutation	Phylop Score	Mutation Taster	PROVEAN (score)	SIFT (score)	CADD (score)	Polyphen-2	MetaSVM
*PAX3*	p.V83G	4.921	Disease causing	Deleterious (−5.671)	Damaging (<0.001)	Deleterious (28.1)	probably damaging	Damaging
*SOX10*	p.W114R	4.612	Disease causing	Deleterious (−11.536)	Damaging (0.001)	Deleterious (25.7)	probably damaging	Damaging
*SOX10*	p.N109S	4.612	Disease causing	Deleterious (−4.183)	Damaging (<0.001)	Deleterious (24.2)	probably damaging	Damaging
*MITF*	p.R214Q	5.952	Disease causing	Deleterious (−3.721)	Damaging (<0.001)	Deleterious (35.0)	probably damaging	Damaging

**Table 4:** Pigmentary abnormalities in WS2 probands with *SOX10* and *MITF* mutations.

Phenotypes	Numbers (%) of WS2 probands		*P*-values
	with *SOX10* mutations	with *MITF* mutations	
Heterochromic iridis	13 (100)	7 (70)	0.068
Premature graying of the hair	1 (8)	4 (40)	0.127
Patchy de-pigmentation of the skin	3 (23)	1 (10)	0.604
Excessive freckles	0 (0)	7 (70)	4.9×10^4^
Total	13 (100)	10 (100)	—
