# Supplementary material for: Molecular etiology and genotype-phenotype correlation of Chinese Han deaf patients with type I and type II Waardenburg Syndrome
Source: Sci Rep. 2016 Oct 19;6:35498. doi: 10.1038/srep35498 (PMC5069774; doi:10.1038/srep35498)
Supplement: Supplementary Information [file srep35498-s1.pdf]

Molecular etiology and genotype-phenotype correlation of Chinese Han deaf patients with type I and type II Waardenburg Syndrome

Lianhua Sun[§1,2,3], Xiaohua Li[§4], Jun Shi[§1,2,3], Xiuhong Pang[5], Yechen Hu[6], Xiaowen Wang[1,2,3], Hao Wu[*1,2,3,7] Tao Yang[*1,2,3]

[1]Department of Otolaryngology–Head and Neck Surgery, Xinhua Hospital, Shanghai Jiaotong University School of Medicine, Shanghai, China

[2]Ear Institute, Shanghai Jiaotong University School of Medicine, Shanghai, China

[3]Shanghai Key Laboratory of Translational Medicine on Ear and Nose Diseases, Shanghai, China

[4]Department of Otology, the First Affiliated Hospital of Zhengzhou University, Zhengzhou, Henan, China

[5]Department of Otorhinolaryngology-Head and Neck Surgery, Taizhou People's Hospital, Jiangsu Province, China

[6]High School Affiliated to Fudan University, Shanghai, China

[7]Department of Otorhinolaryngology-Head and Neck Surgery, Shanghai Ninth People's Hospital, Shanghai Jiaotong University School of Medicine, Shanghai, China

[§]Those authors contributed equally to this work

***Corresponding Authors:**

Tao Yang, Ear Institute, Shanghai Jiaotong University School of Medicine, Shanghai, China. Email: yangtfxl@sina.com, Tel: 86-21-25078893, Fax: 86-21-65152394

Hao Wu, Department of Otolaryngology-Head & Neck Surgery, Shanghai Ninth People's Hospital, Shanghai Jiaotong University School of Medicine, Shanghai, China. Email: wuhao622@sina.cn, Tel: 86-21-55570010, Fax: +86-21-65152394

Supplementary Table S1. Analysis of gender and age as potential confounding effects on the excessive freckle phenotype in WS2 patients with *MITF* and *SOX10* mutations.

	Patient	Mutations	Gender	Age
Patients with *MITF* mutations and excessive freckles	W14-1	p.R110X	F	15
	W16-4	p.X420Qext33	M	27
	W16-3	p.X420Qext33	F	29
	W16-5	p.X420Qext33	M	61
	W18-1	c.710+1G>T	F	31
	W19-1	p.R270X	M	3
	W19-4	p.R270X	M	35
	W19-7	p.R270X	F	23
	W19-8	p.R270X	F	20
	W19-9	p.R270X	M	49
	W19-10	p.R270X	F	51
	W19-11	p.R270X	M	60
	W21-1	p.R255X	M	31
	W44-1	c.494delC	F	8
	W44-2	c.494delC	M	30
	W46-1	p.R255X	M	6
Patients with *MITF* mutations but not excessive freckles	W14-2	p.R110X	F	9
	W46-2	p.R255X	M	39
	D2-1	p.R259X	M	3
	W33-1	p.R214Q	M	3
	W33-3	p.R214Q	F	28
Patients with *SOX10* mutations but not excessive freckles	W8-1	c.1083delG	M	17
	W10-1	c.1074delA	F	19
	W11-1	c.495-496insA	F	18
	W12-1	c.36-54del19bp	F	2
	D680-1	c.400delC	M	1
	W25-1	c.1095delG	M	9
	W47-1	c.690delC	M	1
	W3-1	p.S40X	M	18
	W3-2	p.S40X	F	14
	W4-1	p.Q197X	F	13
	W4-2	p.Q197X	F	39
	W23-1	p.W85X	M	5
	W26-1	c.396-397ins21	M	2
	W17-1	p.W114R	M	1
	W27-1	p.N109S	M	4
***P*-values**[*]			**0.8129**	**0.1559**

[*]The confounding effects of gender and age on the excessive freckle pheontype were tested by logistic regression analysis as independent variables.
